# Sulforaphane Enhances the Ability of Human Retinal Pigment Epithelial Cell against Oxidative Stress, and Its Effect on Gene Expression Profile Evaluated by Microarray Analysis

**DOI:** 10.1155/2013/413024

**Published:** 2013-09-25

**Authors:** Liang Ye, Ting Yu, Yanqun Li, Bingni Chen, Jinshun Zhang, Zhongyang Wen, Bo Zhang, Xiaohong Zhou, Xiaoqing Li, Feng Li, Wei Cao, Zhong Huang

**Affiliations:** ^1^Institute of Biotherapy, Shenzhen University School of Medicine, Nanhai Ave 3688, Shenzhen, Guangdong 518060, China; ^2^Department of Ophthalmology, Dean A. McGee Eye Institute, University of Oklahoma Health Sciences Center, 608 Stanton L. Young Blvd., Oklahoma City, OK 73104, USA

## Abstract

To gain further insights into the molecular basis of Sulforaphane (SF) mediated retinal pigment epithelial (RPE) 19 cell against oxidative stress, we investigated the effects of SF on the regulation of gene expression on a global scale and tested whether SF can endow RPE cells with the ability to resist apoptosis. The data revealed that after exposure to H_2_O_2_, RPE 19 cell viability was increased in the cells pretreated with SF compared to the cell not treated with SF. Microarray analysis revealed significant changes in the expression of 69 genes in RPE 19 cells after 6 hours of SF treatment. Based on the functional relevance, eight of the SF-responsive genes, that belong to antioxidant redox system, and inflammatory responsive factors were validated. The up-regulating translation of thioredoxin-1 (Trx1) and the nuclear translocation of Nuclear factor-like2 (Nrf2) were demonstrated by immunoblot analysis in SF treated RPE cells. Our data indicate that SF increases the ability of RPE 19 cell against oxidative stress through up-regulating antioxidative enzymes and down-regulating inflammatory mediators and chemokines. The results suggest that the antioxidant, SF, may be a valuable supplement for preventing and retarding the development of Age Related Macular Degeneration.

## 1. Introduction

Oxidative stress has been shown to be a major factor in the etiology of age-related macular degeneration (AMD) [1], which is a common cause of visual loss among individuals who are over 65. RPE have been shown to play a crucial role in defenses against photoreceptor damage by absorbing and filtering light, scavenging free radicals, and removing lipids, proteins, and DNA damaged by photo oxidation. The pathology of AMD is thought to be secondary to the degeneration of retinal pigment epithelial (RPE) cells. This is supported by the two early signs of AMD, drusen and lipofuscin, which are formed within RPE [2]. Furthermore, the degeneration of RPE cells is often observed in the early stages of AM before the degeneration of photoreceptors and vision impairments [3]. Due to direct exposure to light [4], high metabolic activity [5], significant oxidative load from the phagocytosis of photoreceptor outer segments [5], and a high proportion of polyunsaturated fatty acids [6], RPE cells are vulnerable to oxidative damage and resulting dysfunction and degeneration [7]. Therefore, protecting RPE cells from photooxidative damage and inflammatory reaction is particularly important in retarding the progression of AMD processes [8].

Sulforaphane (SF), a naturally occurring antioxidant found as a precursor of glucosinolate in broccoli has, over the last several years, emerged as an antiphotoreceptor degeneration agent [9]. Pretreatment of human adult RPE 19 cells with SF provided a powerful and long-term protection against the toxicities of various oxidants and photo-oxidative damage by upregulating the expression of antioxidant and detoxification enzymes and inhibiting inflammatory responses [10]. The extent of photooxidative protection by SF has been shown to correspond to the quantitative induction of phase 2 response enzymes such as NAD(P)H:quinone oxidoreductase and increases in the level of reduced glutathione [9]. Intraperitoneal and oral administration of SF increased the expression of Trx in retinal tissue and upregulated genes with cytoprotective effects against light-induced damage to photoreceptors and RPE in mice [11]. In our previous study, we showed that systemic administration of SF could delay photoreceptor degeneration via inducing the activity of ERKs and up-regulating Trx/TrxR/Nrf2 system in the retinas of *tub/tub* mice [12]. In this study, we aimed to gain further insights into the molecular basis of SF mediated RPE 19 cells against oxidative stress.

## 2. Materials and Methods

### 2.1. Human RPE 19 Cell Culture

 Human RPE 19 cells (ATCC, Manassas, VA) were grown in Dulbecco's modified Eagle's medium (DMEM; Invitrogen, Carlsbad, CA). The medium was supplemented with 2 mM glutamine, 10 IU/mL penicillin, 10 *μ*g/mL streptomycin, and 10% heat-inactivated fetal calf serum (FCS; Invitrogen). Cells were grown in an incubator with a humidified atmosphere of 5% CO_2_ and 95% air at 37°C, and were trypsinized and seeded into 6-well flat-bottomed plates (Falcon, Fort Worth, TX) containing 3 mL of the same medium. After 48 hours of incubation, the density of the cells reached 60 to 70% confluence, and treatments of the cells were then conducted.

### 2.2. Detection of RPE 19 Cell Apoptosis by Flow Cytometry

Cultured RPE 19 cells were pretreated with or without 10 *μ*M SF for 12 hours, then phosphate-buffered saline (PBS) or 400 *μ*M of H_2_O_2_ were prepared in the cell culture medium and added into the cell cultures. RPE 19 cells were collected at 0, 6, 12, and 18 hours after treatments. The cells were collected after trypsin digestion, and apoptosis was determined by flowcytometry using Annexin V-FITC kit (Beckman Coulter, Fullerton, CA) according to the manufacturer's instructions. Briefly, human RPE 19 cells (10^5^/mL) were washed with PBS and resuspended in binding buffer in the dark before staining with 2 *μ*L of annexin V (0.5 *μ*g/mL) and 10 *μ*L propidium iodide (0.6 *μ*g/mL) for 10 min at room temperature. After staining, cells were analyzed immediately using a FACScan flowcytometer (Beckman Coulter, Fullerton, CA) with simultaneous monitoring of green fluorescence (530 nm, 30 nm bandpass filter) for annexin V-FITC and red fluorescence (long-pass emission filter that transmits light > 650 nm) for propidium iodide. A total of 30,000 events were collected and analyzed.

### 2.3. Microarray Analysis of mRNA of SF-Treated Human RPE 19 Cells

#### 2.3.1. RNA Sample Preparations

Cultured RPE 19 cells were pretreated with or without 10 *μ*M SF for 12 hours, quadruplicate cultures of RPE 19 cells were collected at 0, 6, and 12 hours after treated with 400 *μ*M of H_2_O_2_. RNA isolations from cultured RPE 19 cells were carried out using Trizol reagent (Invitrogen, Carlsbad, CA) according to the manufacturer's instruction and purified with an RNA cleanup kit (Qiagen; Valencia, CA). The concentration of RNA was determined with a Nanodrop scanning spectrophotometer, and then qualitatively assessed for degradation using the ratio of 28 : 18 s rRNA obtained from a capillary gel electrophoresis system (Agilent 2100 Bioanalyzer, Agilent Technologies).

#### 2.3.2. Labeling and Hybridization

cDNA synthesis, hybridization, and staining were performed as specified by Affymetrix (Santa Clara, CA). Briefly, 2.8 *μ*g of total RNA was primed with T7-oligo-dT and reversily transcribed with SuperScript II, followed by the production of double-stranded cDNA with *E. coli* DNA polymerase. cRNA was transcribed in vitro from the T7 promoter using a biotinylated ribonucleotide analog and then fragmented to approximately 100 nt. cRNA was hybridized to Human Genome U133 Plus 2.0 GeneChip microarrays. These arrays contain probes for approximately 47,000 transcripts in the human genome. GeneChips were washed and stained using an Affymetrix automated GeneChip 450 fluidics station and scanned with an Affymetrix 3000 7G scanner.

#### 2.3.3. Normalization of Array Data

All array preprocessing was performed in the R/Bioconductor Package, “Affy.” The raw Affymetrix Perfect Match probes were normalized by the RMA method combined with median-polish. The marginal data distributions were adjusted through quantile normalization. The resulting normalized values were imported into BRB ArrayTools (Biometric Research Branch, National Cancer Institute) where they were log transformed. Genes were filtered by using the “Log Expression Variation Filter” to screen out genes that are not likely to be informative, based on the variance of each gene across the arrays. The filter was set to exclude genes that fell below the 75th percentile of gene variance. We identified genes that were differentially expressed between any two classes (0 to 6, and 0 to 12) by using a multivariate permutation test [13]. We used the multivariate permutation test to provide a median false discovery rate (FDR) of 5% (80% confidence). The test statistics used were random variance *t*-statistics for each gene [14]. Although *t*-statistics were used, the multivariate permutation test is nonparametric and does not require the assumption of Gaussian distributions. Data were exported to Excel where averages of the classes were used to calculate expression ratios. Genes that were differentially expressed (<5% FDR) and simultaneously had a ratio of 2-fold or larger were used in further analyses.

### 2.4. Real-Time Quantitative Reverse Transcription (qRT-PCR)

Real-time quantitative reverse transcription-polymerase chain reaction (qRT-PCR) was performed as previously described [15]. SYBR Green was used as a fluorescent detection dye, the qRT-PCR performed in a Bio-Rad iCycler (Hercules, CA). Eight genes were chosen for the confirmation on the basis of their functional importance. The RNA was harvested from RPE 19 cells under similar treatments as used for the microarray study. Fold changes (mean ± SD) were calculated from four independent replicate groups.

### 2.5. Semiquantitative Reverse Transcription (RT)-PCR Analysis

Performance of sq-RT-PCR was as previously described [15]. Briefly, total mRNA was extracted by Trizol reagent, and first-strand cDNA was synthesized with the kit of SuperScript First-Strand Synthesis System (Invitrogen, Carlsbad, California), according to the manufacturer's instructions. The same primer pairs (Table 1) used for real-time qRT-PCR were also used for sqRT-PCR.

### 2.6. Western Blot Analysis

Western blot analysis was performed as described previously [16]. RPE 19 Cells were treated the same as described for microarray analysis, collected and lysed in buffer containing 15 mM Tris-HCl (pH 7.6), 150 mM NaCl, 0.1 mM EDTA, 10% glycerol, 1% Triton X-100, and protease inhibitor cocktail (Sigma; St. Louis, MO). Supernatants were obtained after centrifugation at 20,000 ×g in a microcentrifuge for 15 min at 4°C. For Nrf2 nuclear translocation studies, nuclear proteins were extracted by Qiagen Q protein Cell compartment Kit according to the manufacturer's instructions (Qiagen; Valencia, CA). Protein concentrations were determined with a BCA protein assay kit (Pierce; Rockford, III). The immunoreactive proteins were detected using enhanced chemiluminescence reagents (Amersham, Piscataway, N.J.) and a LumiImager (Fujuifilm Medical Systems USA Inc., Stamford, Conn.). Loading controls were carried out by probing with anti-*β*-actin for Trx1. Antibodies against *β*-actin and Nrf2 were from Santa Cruz Biotechnology, Inc. (Santa Cruz, CA) and anti-Trx1 was from Abcam (Cambridge MA).

### 2.7. Statistical Analysis

Results are expressed as mean ± SD. Statistical differences between the control and different time points were determined by using one-way analysis of variance and the *t*-test. A *P* values < 0.05 was considered significant.

## 3. Results

### 3.1. Enhancing the Ability of RPE 19 Cells to Resist

**Figure 1:**
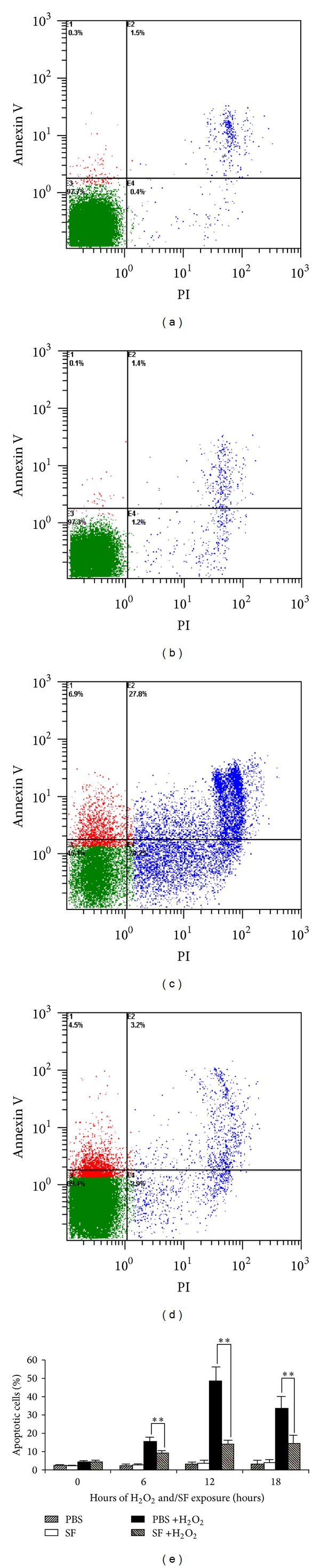
Sulforaphane (SF) endows the ability of antioxidative stress to RPE 19 cells from H_2_O_2_ induced apoptosis. Flow cytometry images and histogram presentations show cultured RPE 19 cells treated with or without Sulforaphane (SF) and H_2_O_2_. (a) Controls treated with PBS, or (b) SF only; (c) cells pretreated with PBS or (d) SF for 12 hours and then incubated with 400 *μ*M H_2_O_2_ for 12 hours. Quadrant E1 (in cytometry images (a), (b), (c), and (d)) indicates cells undergoing apoptosis; E2 represents cells that died of secondary necrosis; E3 represents live cells; and E4 represents necrotic cells. The bar graph (e) shows RPE 19 cell apoptotsis under four different treatment conditions at four time points. The data came from four biological replicates, and mean ± SD of apoptotic rate are presented. Significant difference is indicated by asterisks; ** represents *P* < 0.01.

To test whether SF could increase the ability of RPE 19 cells to withstand oxidative stress, the dose dependent experiments were conducted. The experiments showed that 10 *μ*M SF was the optimal amount of SF to endow the ability of antioxidative stress for RPE 19 cells (see Figure 1 in Supplementary Material available online at http://dx.doi.org/10.1155/2013/413024). To asses the antiappototic ability endowed by SF in RPE 19 cells, the cells were pretreated with 10 *μ*M SF or PBS (control) for 12 hours, and then 400 *μ*M H_2_O_2_ was to induce cellular apoptosis. Representative flow cytometry images showed that the apoptotic rates in cultured RPE 19 cells did not differ between the groups treated with PBS or SF alone (Figures 1(a) and 1(b)). However, in those cultures treated with H_2_O_2_, RPE cell viability was much higher in the cells pretreated with SF than in the PBS treated cultures. (Figures 1(c) and 1(d)). The data from four independent experiments, compiled and analyzed quantitatively, are shown (Figure 1(e)). By comparison of the different time points for H_2_O_2_ treatment, it is seen that the peak of cellular apoptosis occurs at 12 hours. The percentage of apoptotic RPE cells was 48% in the group treated with PBS and H_2_O_2_, whereas it was only 14% in the cultures treated with SF and H_2_O_2_. Statistical analysis showed that the *P* value was less than 0.01 between the groups with and without SF treatment. The percentage of apoptotic cells increases from 6 to 12 hours (19% to 48%) but decreases after 18 hours (35%) with H_2_O_2_. (*P* value < 0.01 (Figure 1(e))). This decrease at 18 hours is most likely due to cells already having gone through apoptosis and simply not present for detection at this time point. The antioxidative effects of SF against oxidative stress were observed at 6 hours; reaching a maximum at the 12-hour time point and were slightly reduced at 18 hours.

### 3.2. Identification of Genes Responsive to SF Treatment by Microarray Analysis

To investigate the antiapoptotic mechanism by SF in human RPE 19 cells, the cells were exposed to 10 *μ*M SF for up to 12 hours, and total RNA samples were collected at 0, 6, and 12 hours after treatment with SF and analyzed by microarray analysis. The genes responsive to SF treatment were selected by the multivariate permutation test [13]. All genes that were at least two fold differentially expressed among any of the 3 treatment time points were designated as “hypervariable genes” and input into Affymetrix NetAffx (Affymetrix, Santa Clara, CA) for full annotation. These genes were further refined by eliminating genes with no symbol and with expression less than 50 (~background). From the 47,000 genes in the array, 69 genes were selected as “hypervariable genes.”

**Figure 2:**
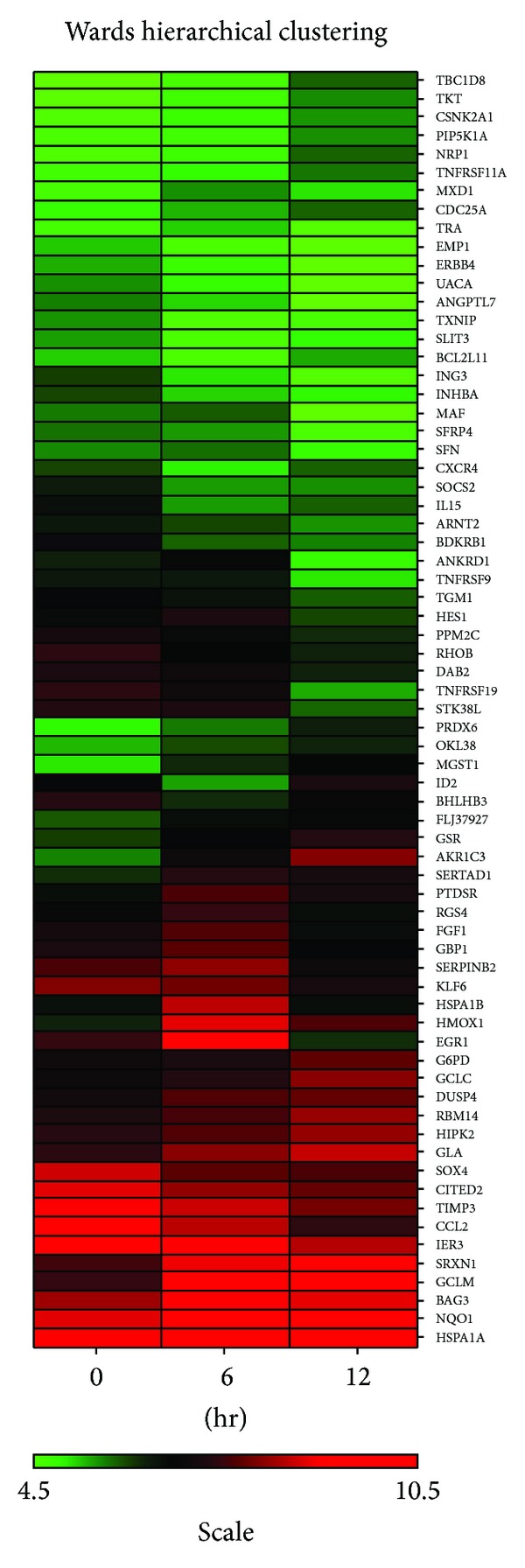
Wards Hierarchical Clustering Analysis of the gene expression at 0, 6, and 12 hours after treated with 400 *μ*M of H_2_O_2_ in RPE 19 cells pretreated with 10 *μ*M SF for 12 hours. Green represents low score of gene expressions; red represents high score of gene expressions. Total of 69 genes were in the records.

The normalized, logged, and averaged expression values of the selected 69 genes from four treatments were input into SpotFire DecisionSite 9.0 software (TIBCO Software, Palo Alto, CA) to create an expression heatmap. The image of the heatmap was created by hierarchical clustering function in SpotFire with Ward's method, which represents the changes and the descriptions of these genes. Compared to 0 h, 31 of 69 genes were upregulated, and the rest of 38 were downregulated in RPE cells at 6 h and 12 h treated with SF. Green represents a lower level of gene expressions, and red represents a higher level of gene expressions (Figure 2).

Detailed information about these genes is presented (Table 2). These sixty-nine genes that had known annotation information belonged to various categories, including genes described as antioxidant, detoxification, cell growth regulation, antiapoptosis, apoptosis, angiogenesis, immunoregulatory, inflammatory response, and signal transduction. For each gene, ratios against 0 hour control from four treatments were used in clustering analysis. Thus, genes that clustered together have similar dynamic patterns. The dendrogram (not shown) was used to group genes into 11 distinct clusters (Table 2).

### 3.3. Confirmation of Candidate Genes from Microarray mRNA Expression Patterns by Real-Time qRT-PCR and sqRT-PCR

To confirm the gene array expression data, we performed qRT-PCR and sqRT-PCR. Based on their functional importance and the research project relevance, eleven of the 69 hypervariable genes were selected for confirmation microarray mRNA results. The expression patterns of eight genes were confirmed by qRT-PCR and sqRT-PCR. They are (1) NAD(P)H:quinone oxidoreductase (NQO1), (2) sulfiredoxin 1 homolog (SRXN1), (3) glutamate-cysteine ligase modifier subunit (GCLM), (4) thioredoxin interacting protein (TXNIP), (5) chemokine (C-C motif) ligand 2 (CCL2), (6) bradykinin receptor B1 (BDKRB1), (7) thioredoxin 1 (Trx1), and (8) transcription factor NF-E2-related factor 2 (Nrf2).

**Figure 3:**
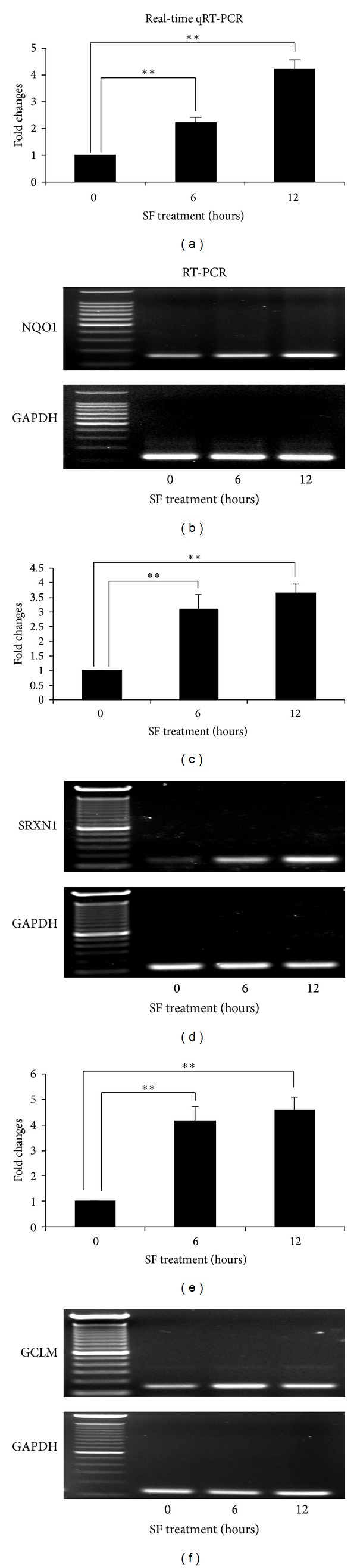
SF induces and increase in the transcripts for NQO1, SRXN1, and GCLM in the RPE 19 cells over time. qRT-PCR was used to show progressive increases over time in the amount of mRNA for NQO1 ((a), (b)), SRXN1 ((c), (d)), and GCLM ((e), (f)) following addition of SF to the cultures. GADPH was used as a loading control. The data for qRT-PCR were normalized with GADPH. The fold changes were calculated from the groups of 6 and 12 hours of SF treatment versus the 0 hour SF treatment. Data are presented as mean ± SD (*n* = 5 in each group). Significant differences are indicated by asterisks, ** represents *P* < 0.01.

**Figure 4:**
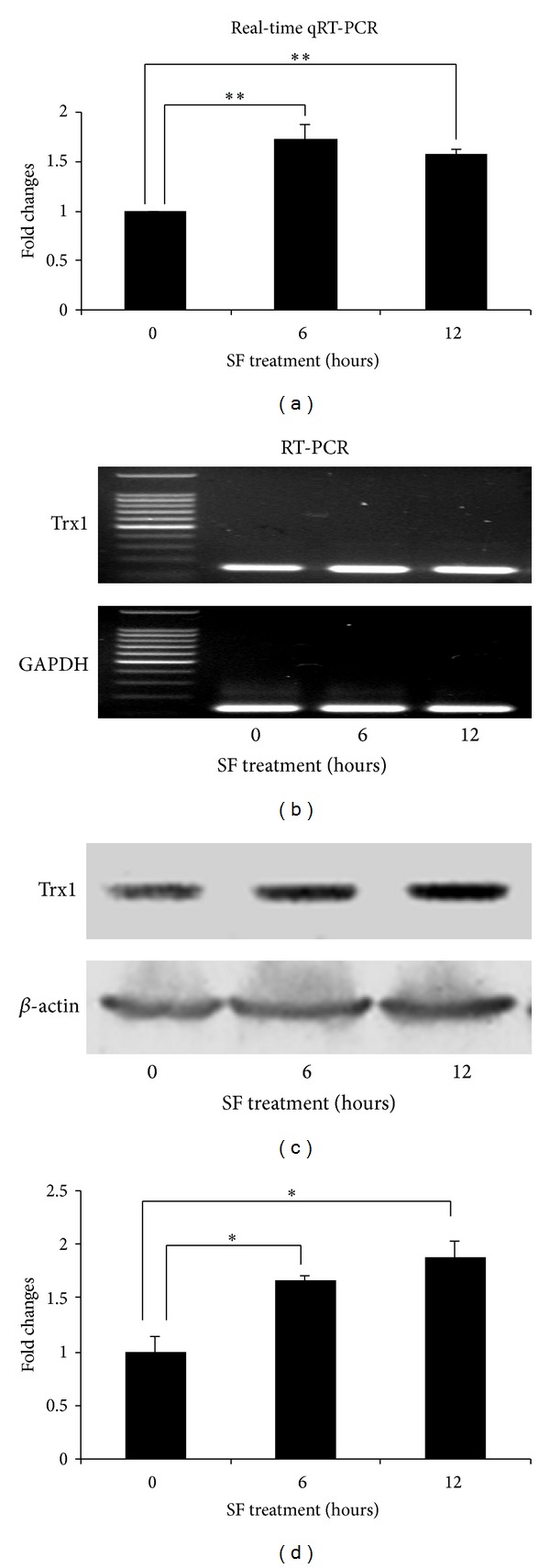
SF increases expression of Trx1 in RPE19 cells. The amount of Trx1 mRNA was shown by qRT-PCR and sqRT-PCR ((a) and (b)) to increase at least 6 hours and remained higher than control levels at 12 hours. However, Western blot data ((c) and (d)) showed a progressive increase in the amount of Trx1 protein from 6 h to 12 h time points. Data are presented as mean ± SD (*n* = 5 in each group of real-time qRT-PCR; *n* = 3 in each group of immunoblots). Significant differences are indicated by asterisk; * represents *P* < 0.05, and ** represents *P* < 0.01.

The antioxidant enzymes upregulated by SF in the cultured RPE 19 cells included: NQO1, a reductase, which catalyzes the beneficial two-electron reduction of quinines to hydroquinones (Figures 3(a) and 3(b)); SRXN1, a redox protein, which generates cysteine from cysteine sulfinic acid; (Figures 3(c) and 3(d)); GCLM is modulatory subunit of glutamate-cysteine ligase that catalyzes the first, and rate-limiting, step in glutathione (GSH) biosynthesis (Figures 3(e) and 3(f)); and Trx1 (Figures 4(a) and 4(b)) a multifunctional redox regulator, which not only serves as a disulfide-reducer for oxidized cysteine groups on the proteins, but also is involved in various intracellular signal transduction pathways [17]. For different time points of SF treatment, the expressional levels of SRXN1, GCLM, and Trx1 only had a small variations from 6 to 12 hours, they are 3.1 : 3.6, 4.1 : 4.6, and 1.72 : 1.57, respectively, whereas NQO1 was almost double of its expression level from 6 hours to 12 hours with SF treatment (2.2 : 4.2). The expression patterns of NQO1, SRXN1, GCLM, and Trx1 detected by qRT-PCR were basically consistent with the results of microarray analysis (Table 3).

**Figure 5:**
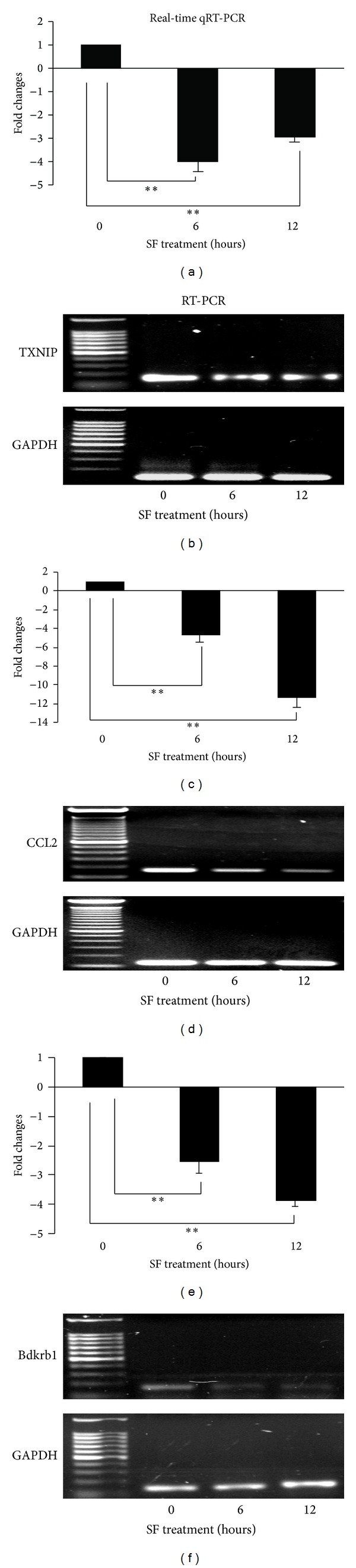
SF down regulates expression of TXNIP, CCL2 and Bdkrb1 in RPE 19 cells. The amount of mRNA for TXNIP ((a), (b)), CCL2 ((c), (d)), and Bdkrb1 ((e), (f)) were determined by qRT-PCR ((a), (c), and (e)) and by sqRT-PCR ((b), (d), and (f)). GADPH was used as a loading control for normalization. Data are presented as mean ± SD (*n* = 5 in each group). Significant differences are indicated by asterisk, ** represents *P* < 0.01.

The transcriptional levels of TXNIP, CCL2, and BDKRB1 (Figure 5) were down regulated after treatment with SF in the RPE 19 cells. The peak inhibition of TXNIP transcription by SF was at 6 hours; however, the peak inhibition of expression of CCL2 and BDKRB1 occurred at 12 hours with SF. The microarray and qRT-PCR profiles of the expression of these three genes quantified were similar (Table 3). Both CCL2 and BDKRB1are inflammatory responsive factors; however, TXNIP is an endogenous biological inhibitor of Trx1.

### 3.4. Upregulated Trx1 and Downregulated TXNIP by SF

Our microarray data showed a slight increase in the transcriptional level of Trx1 in the RPE 19 cells treated with SF for 6 and 12 hours compared to the group treated with SF for 0 hour, respectively (1.46 : 1 and 1.53 : 1). The expression of Trx1mRNA detected by sqRT-PCR in the groups with or without SF treatment showed a pattern similar to the microarray analysis (Figure 4(b)). By qRT PCR, the fold change from 0 hour to 6 hour was 1.72 : 1 and from 0 hour to12 hour was 1.57 : 1 (Figure 4(a) and Table 3). Due to only a slight increment of the Trx1 transcriptional level in SF treated group compared to control group, immunoblot analysis was applied to look at the translational level of Trx1 under the conditions of SF treated for 0, 6, and 12 hours. As shown in Figure 4(c), the protein expression level was indeed increased in the cells treated with SF compared to untreated RPE 19 cells. Densitometric analysis showed that the amount of Trx1 protein in the cells treated with SF for 6 hours was 65% higher than that in the control cells whereas the cells treated with SF for 12 hours had 87 percent more Trx1 than untreated cells (Figures 4(c) and 4(d)).

A surprising result seen in the microarray data (Figure 2) and confirmed by sqRT-PCR and qRT-PCR (Figures 5(a) and 5(b)) was the expression of TXNIP, an endogenous inhibitor of Trx1, dramatically inhibited by SF. qRT-PCR showed that 6 hours with SF resulted in about a four fold inhibition of TXNIP and this had only decreased to about 3 fold after 12 hours exposure to SF (Figure 5(a)). These results suggest that SF might coordinate the expression of Trx1 and TXNIP to increase the activity of Trx1 in RPE 19 cells.

### 3.5. SF Mediates the Nuclear Translocation of Nrf2

**Figure 6:**
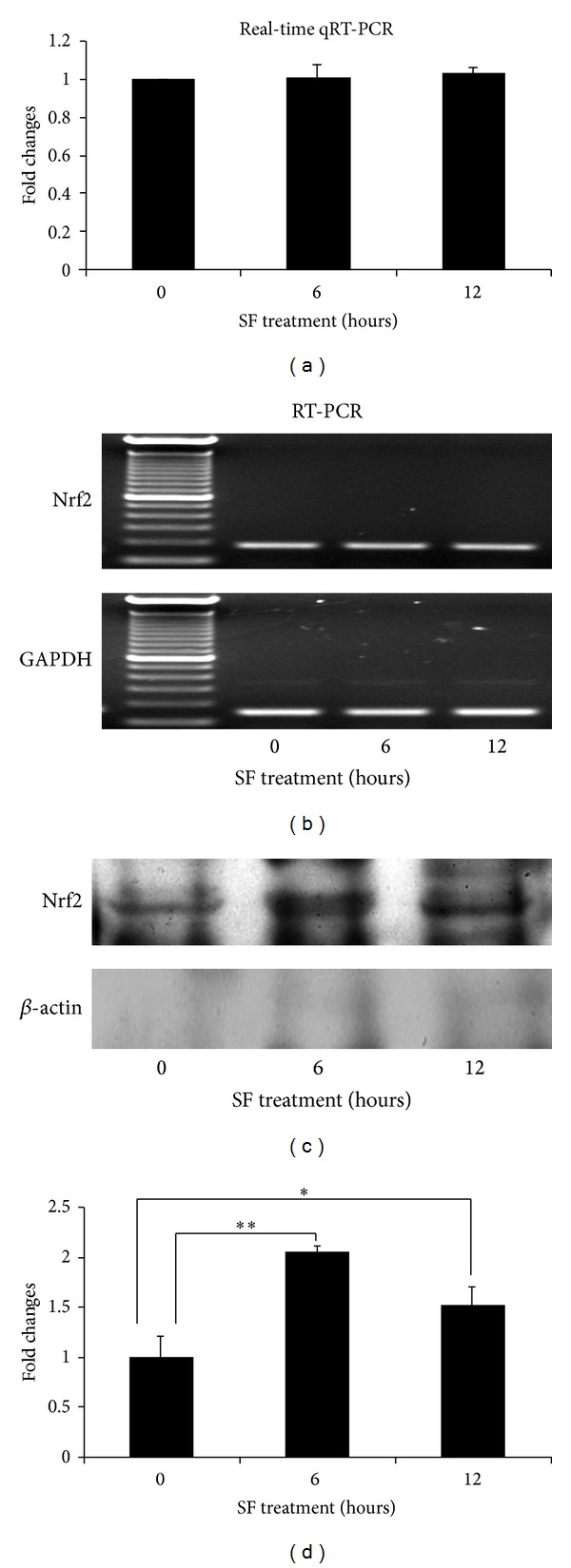
Histogram presentation of fold change of Nrf2 quantified by real-time qRT-PCR (a) and images of sqRT-PCR with GADPH as loading control (b). Nuclear extractions of the cultured RPE 19 cells treated with or without SF were probed with anti-Nrf2 (c). Densitometric analysis of western blots (d). The amount of Nrf2 in the nuclear fraction increased in the presence of SF compared to untreated cells. Data are presented as mean ± SD (*n* = 5 in each group of real-time qRT-PCR; *n* = 3 in each group of immunoblots). Significant differences are indicated by asterisk; * represents *P* < 0.05, and ** represents *P* < 0.01.

Several genes, which contain consensus antioxidant response element (ARE) promoters, such as NQO1, GCLM, and Trx1 have been shown to be upregulated by SF in the microarray experiments and confirmed by sqRT-PCR and qRT-PCR. Gene induction through ARE involves a process that is dependent on the nuclear factor-erythroid 2p45-related factor 2 (Nrf2). Surprisingly, in the microarray experiments, Nrf2 did not show an elevated transcriptional level following SF treatment and this result was confirmed by sqRT-PCR and qRT-PCR (Figures 6(a) and 6(b)). To test whether the up regulation of NQO1, GCLM, and Trx1 was due to the activation of Nrf2, nuclear extracts from the RPE 19 cells treated with or without SF were subjected to immunoblot analysis with an antibody against Nrf2. Compared to control, a heavy immunostaining of Nrf2 was found in the lane of treated with SF for 6 hours, after 12 hours of SF treatment, the amount of Nrf2 in the nuclei was less than that of 6 hours, but still higher than that the group of control (Figures 6(c) and 6(d)). The same membranes were stripped and probed with anti-*β* actin (Figure 6(c)). The absence of this cytoplasmic protein indicates the purity of the nuclear extractions from RPE 19 cells.

## 4. Discussion

We have demonstrated for the first time that SF significantly inhibits H_2_O_2_ induced human RPE cell apoptosis. This result is meaningful because the accumulated evidence indicates that RPE 19 cells impaired by oxidative stress, could alter the extra cellular environment of photoreceptors cells. This would include changes in metabolic products and nutrient transport [18], neurotrophic factor production [19], and clearance of molecules damaged by photo-oxidation [20], and could contribute to photoreceptor cell degeneration [21]. The gene expression profile analysis in this study showed that antioxidative effects of SF on the survival of human RPE cells occur mainly through the induction of the expression of antioxidant genes and the inhibition of anti-inflammatory responsive genes. Therefore, SF could be a useful dietary supplement for the prevention of photo-oxidative stress-related retinal diseases including AMD.

To study the mechanism underlying the SF mediated antioxidative effect of RPE 19 cells, a DNA microarray approach was used to analyze the variations in gene expression in RPE 19 cells in response to SF. Transcriptional levels of 69 genes were regulated by SF in RPE 19 cells. The genes affected code for proteins whose activities are involved in a variety of cellular processes including antioxidation, detoxification, cell growth regulation, antiapoptosis, apoptosis, angiogenesis, immunoregulatory, inflammatory response and signal transduction. The change in expression of eight of the 69 hypervariable genes was confirmed by qRT-PCR, and sqRT-PCR. The specific genes are NQO1, SRXN1, GCLM, TRX1, NRF2, BDKRB1, TXNIP, and CCL2.

The genes which were upregulated in RPE cells treated with SF were NQO1, SRXN1, GCLM, and TRX1. They all code for important antioxidant and detoxification enzymes, play crucial roles in cellular antioxidative stress, antiapoptosis, detoxification, anticarcinogenesis and signal transduction. NAD(P)H:quinone oxidoreductase (NQO1) is involved in the cellular defense against oxidative stress via direct reduction and detoxification of highly reactive quinines [22]. In addition, NQO1 has been shown to stabilize p53 in response to DNA-damaging stimuli [23]. Sulfiredoxin 1 (Srxn1), a small thiol containing protein, acts as a regulator of the redox-activated thiol switch in cells by catalyzing deglutathionylation of specific proteins in response to reactive oxygen species [24]. Dr. Hardingham studies discovered that Srxn1 contains a AP-1 site within its ARE, and shows that the gene can be induced by Nrf2 activator CDDO-TFEA [25–27]. The modulatory subunit of glutamate-cysteine ligase (GCLM) is a limiting factor for forming glutamate-cysteine ligase (GCL) [28]; an enzyme which catalyzes the first and rate-limiting step in glutathione (GSH) biosynthesis. GSH serves as a reductant in numerous biochemical reactions which counteract oxidative events and protect protein thiol groups [29]. Thioredoxin 1 (Trx1) catalyzes the reversible reduction of disulfides by utilizing its cysteinyl residues in the Cys-X-X-Cys active site. Through its function as a scavenger of reactive oxygen species, Trx1 plays a crucial role in cellular defense against various oxidative stresses [30], inflammatory responses [31], and light-induced photoreceptor degeneration [32]. Upregulation of these four important endogenous antioxidant proteins by SF in human RPE 19 cells provides direct evidence that SF could stimulate a wide range of antioxidant enzymatic activities in RPE cells in vivo.

Thioredoxin interacting protein (Txnip) is one of the three proteins down regulated by SF in RPE cells and confirmed by qRT-PCR and sqRT-PCR. Txnip, a ubiquitously expression protein, has been demonstrated to bind to thioredoxin and inhibit its activity [33]. Down-regulation of the expression of Txnip without affecting thioredoxin expression and leading to a net increase in the activity of thioredoxin has also been shown to occur in vascular endothelial cells, smooth muscle cells, and cardiomyocytes [34]. Thus, the antioxidative capacity of the RPE cells is increased by Txnip downregulation whereas they can be made more susceptible to oxidative stress and apoptosis by Txnip upregulation [35].

By decreasing cellular redox capacity, the proteins and peptides that contain thiol group of cysteinyl side chains will be susceptible to a number of oxidative modifications, such as the formation of inter- or intramolecular disulfides between proteins or low-molecular-weight peptide (glutathione) thiols and oxidization of sulfenic to sulfinic and to sulfonic acid [36]. These modifications result in the changes of structures and functions of numerous proteins that contain cysteines, and the alterations of their catalytic activities and protein-protein interaction affinity of the proteins. It is worth noting that the reductive ability of the redox system in RPE 19 cells treated with SF was upregulated by increasing the expression of two thiol reductases Srxn1 and Trx1 and by decreasing the expression of a Trx1 endogenous biological inhibitor Txnip. These indicate that the intracellular redox reduction activity is strongly stimulated by SF and imply that the redox system plays an important role in the defense against H_2_O_2_ induced RPE 19 cell apoptosis.

It has been shown that oxidative stress and inflammation are deeply interrelated, and each may cause the other [37]. The upregulation of antioxidant enzymatic activities and down regulation of inflammatory reactions, such as inhibiting the production of chemokines and inflammatory mediators, will coordinately provide a against oxidative stress and inflammation in RPE cells. Chemokine (C-C motif) ligand 2 (CCl2), a member of the CC chemokine family, has been demonstrated to play an important role in the initiation and progression of inflammation [38]. MCP-1 is upregulated in a variety of inflammatory diseases such as atherosclerosis and rheumatoid arthritis [39]. Upon inflammation, MCP-1 recruits and activates monocytes, macrophages, memory T lymphocytes, and natural killer (NK) cells to the site of inflammation [38], which is involved in various pathophysiologic conditions such as inflammation, trauma, burns, shock, and allergy. Increases in Bdkrb1 have been demonstrated to be associated with the production of inflammatory mediators and stimulation of inflammatory cells. In our experiments, microarray analysis, qRT-PCR, and sqRT-PCR show that SF treated RPE 19 cells dramatically inhibited the expression of MCP-1 and Bdkrb1, which indicates that SF might also act by inhibiting the inflammatory response in human RPE cells to reduce apoptosis.

NQO1, GCLM, and Trx1 contain a cis-acting antioxidant response element (ARE) within the regulatory region of their genes [40]. Upon stimulation, Nrf2, a basic leucine zipper (bZIP) transcription factor, is translocated into the nuclei of the cells and forms a heterodimer with either Maf, FosB, c-Jun, and JunD, which then upregulate the expression of genes which contain an ARE element, such as Srxn1 [25–27, 41]. In this study, an increased expression of Nrf2 was not detected in RPE 19 cells treated with SF but a dramatic increase in the nuclear translocation of Nrf2 was found in the nuclear extractions. This suggests that the increase in these three antioxidant genes in SF treated RPE cells is Nfr2 dependent. Our previous results have shown that the regulation of Nrf2 by SF is through stimulation of the activity of extracellular signal-regulated kinases in mouse retinas [12]. Additional data will be needed to define the mechanisms by which SF regulates the expression of Txnip, MCP-1, and Bdkrb1 in RPE 19 cells.

In summary, microarray analysis revealed significant changes in the transcriptional levels of 69 genes in human RPE 19 cells after treated with SF. The genes are involved in a variety of cellular processes such as antioxidation, detoxification, cell growth regulation, antiapoptosis, immuno-regulation, inflammation, and signal transduction. SF endows the ability of antioxidative stress to RPE 19 cells against H_2_O_2_ induced cell apoptosis. This antioxidative effects is mediated by upregulation of antioxidant related genes, such as NQO1, SRXN1, GCLM, and Trx1 and by down regulation of inflammatory responsive genes including CCL2, Bdkrb1, and Txnip. SF appears to act through Nrf2 regulation of ARE containing genes such as NQO1, GCLM, and Trx1.

## Supplementary Material

The antioxidative effects of different concentrations of SF in RPE 19 cells is provided in Supplementary Figure1. RPE 19 cells were pretreated with various concentrations of SF (0 *µ*M, 1 *µ*M, 10 *µ*M, 50 *µ*M, respectively) for 12 hours, then PBS or 400 *µ*M of H2O2 were added into the cell cultures for 12 hours. Flow cytometry analysis showed that 10 *µ*M SF was the optimal amount of SF to endow the ability of antioxidative stress for RPE 19 cells.

## Figures and Tables

**Table 1:** List of oligonucleotides and products size for real-time quantitative PCR and semi-quantitative RT-PCR.

Accession No.	Gene	Forward	Reverse	Product size (bp)
NM_000903	NQO1	5′AAGGATGGAAGAAACGCCTGGAGA	5′ GGCCCACAGAAAGGCCAAATTTCT	156
NM_080725	SRXN1	5′ CCCATCGATGTCCTCTGGATCAAA	5′ AGGTACACCCTTAGGTCTGAGAGA	155
NM_002061	GCLM	5′ CTGCTGTGTGATGCCACCAGATTT	5′ GTGCGCTTGAATGTCAGGAATGCT	145
NM_006472	TXNIP	5′ TTGGCAGCAGATCAGGTCTAAGCA	5′ AGAGGAGTTGTTGGGCTCTCCAAT	166
NM_002982	CCL2	5′ TCGCTCAGCCAGATGCAATCAATG	5′ TCTCCTTGGCCACAATGGTCTTGA	156
NM_000710	BDKRB1	5′ CTGCACAGAGTGCTGCCAACATTT	5′ GCAAGCCCAAGACAAACACCAGAT	166
NM_006164	NRF2	5′ AACTACTCCCAGGTTGCCCACATT	5′ AGCAATGAAGACTGGGCTCTCGAT	174
NM_003329	TRX1	5′ GGACGCTGCAGGTGATAAACTTGT	5′ GCAACATCCTGACAGTCATCCACA	153
NM_002046	GAPDH	5′ TCTCCTCTGACTTCAACAGCGACA	5′ CCCTGTTGCTGTAGCCAAATTCGT	126

**Table 2:** Hypervariable genes responsive to SF treatment in cultured human RPE cells*.

Accession no.	Gene symbol	Description	Cluster order	Cluster
AI034387	TBC1D8	TBC1 domain family, member 8 (with GRAM domain)	1	1
AU152969	TKT	Transketolase (Wernicke-Korsakoff syndrome)	2	1
AI298887	CSNK2A1	Casein kinase 2, alpha 1 polypeptide	3	1
BF515595	PIP5K1A	Phosphatidylinositol-4-phosphate 5-kinase, type I, alpha	4	1
BE620457	NRP1	Neuropilin 1	5	1
AW026379	TNFRSF11A	Tumor necrosis factor receptor superfamily,	6	1
		Member 11a, NFKB activator		
AW071793	MXD1	MAX dimerization protein 1	7	1
AY137580	CDC25A	Cell division cycle 25A	8	1
M15565	TRA	T cell receptor alpha locus	9	2
BC017854	EMP1	Epithelial membrane protein 1	10	2
AW772192	ERBB4	V-erb-a erythroblastic leukemia viral	11	2
		Oncogene homolog 4 (avian)		
AW264269	UACA	Uveal autoantigen with coiled-coil	12	2
		Domains and ankyrin repeats		
NM_021146	ANGPTL7	Angiopoietin-like 7	13	2
NM_006472	TXNIP	Thioredoxin interacting protein	14	2
BC032027	SLIT3	Slit homolog 3 (Drosophila)	15	2
AF455755	BCL2L11	BCL2-like 11 (apoptosis facilitator)	16	2
AF161419	ING3	Inhibitor of growth family, member 3	17	2
M13436	INHBA	Inhibin, beta A (activin A, activin AB alpha polypeptide)	18	2
NM_005360	MAF	V-maf musculoaponeurotic fibrosarcoma	19	2
		Oncogene homolog (avian)		
NM_003014	SFRP4	Secreted frizzled-related protein 4	20	2
X57348	SFN	Stratifin	21	2
AJ224869	CXCR4	Chemokine (C-X-C motif) receptor 4	22	3
NM_003877	SOCS2	Suppressor of cytokine signaling 2	23	3
NM_000585	IL15	Interleukin 15	24	3
NM_014862	ARNT2	Aryl-hydrocarbon receptor nuclear translocator 2	25	3
NM_000710	BDKRB1	Bradykinin receptor B1	26	3
NM_014391	ANKRD1	Ankyrin repeat domain 1 (cardiac muscle)	27	3
NM_001561	TNFRSF9	Tumor necrosis factor receptor superfamily, member 9	28	3
NM_000359	TGM1	Transglutaminase 1 (K polypeptide epidermal type I,	29	4
		Protein-glutamine-gamma-glutamyl transferase)		
BE973687	HES1	Hairy and enhancer of split 1, (Drosophila)	30	4
NM_018444	PPM2C	Protein phosphatase 2C, magnesium-dependent,	31	4
		Catalytic subunit		
AI263909	RHOB	Ras homolog gene family, member B	32	4
N21202	DAB2	Disabled homolog 2, mitogen-responsive	33	4
		Phosphoprotein (Drosophila)		
BF432648	TNFRSF19	Tumor necrosis factor receptor superfamily, member 19	34	4
AW779556	STK38L	Serine/threonine kinase 38 like	35	4
N55072	PRDX6	Peroxiredoxin 6	36	5
NM_013370	OKL38	Pregnancy-induced growth inhibitor	37	5
AV705233	MGST1	Microsomal glutathione S-transferase 1	38	5
NM_002166	ID2	Inhibitor of DNA binding 2, dominant negative	39	6
		Helix-loop-helix protein		
AB044088	BHLHB3	Basic helix-loop-helix domain containing, class B, 3	40	6
AA173223	FLJ37927	CDC20-like protein	41	6
AI888037	GSR	Glutathione reductase	42	6
AB018580	AKR1C3	Aldo-keto reductase family 1, member C3	43	6
		(3-alpha hydroxysteroid dehydrogenase, type II)		
BC002670	SERTAD1	SERTA domain containing 1	44	7
AK021780	PTDSR	Phosphatidylserine receptor	45	7
BC000737	RGS4	Regulator of G-protein signalling 4	46	7
X59065	FGF1	Fibroblast growth factor 1 (acidic)	47	7
NM_002053	GBP1	Guanylate binding protein 1,	48	7
		Interferon-inducible, 67 kDa		
NM_002575	SERPINB2	Serpin peptidase inhibitor, clade B	49	7
		(ovalbumin), member 2		
BG250721	KLF6	Kruppel-like factor 6	50	7
NM_005346	HSPA1B	Heat shock 70 kDa protein 1B	51	7
NM_002133	HMOX1	Heme oxygenase (decycling) 1	52	7
AI459194	EGR1	Early growth response 1	53	7
NM_000402	G6PD	Glucose-6-phosphate dehydrogenase	54	8
NM_001498	GCLC	Glutamate-cysteine ligase, catalytic subunit	55	8
NM_001394	DUSP4	Dual specificity phosphatase 4	56	8
NM_006328	RBM14	RNA binding motif protein 14	57	8
AW300045	HIPK2	Homeodomain interacting protein kinase 2	58	8

*Genes are in the same order as presented in Figure 2.

**Table 3:** Fold changes of gene expression between microarray/real-time quantitative PCR.

Accession no.	Gene symbol	Fold change against 0 hour		
		0	6	12
NM_000903	NQOl	1	2.02/2.22	4.2/4.34
NM_080725	SRXN1	1	3.04/3.31	3.87/3.77
NM_002061	GCLM	1	4.94/4.43	4.86/4.71
NM_003329	TRX1	1	1.46/1.72	1.53/1.57
NM_006472	TXN1P	1	0.28/0.26	0.32/0.33
NM_002982	CCL2	1	0.24/0.23	0.09/0.09
NM_000710	BDKRB1	1	0.42/0.40	0.36/0.25
NM_006164	NRF2	1	1.02/1.04	0.98/1.02

## References

[1] Beatty S, Koh H-H, Phil M, Henson D, Boulton M (2000). The role of oxidative stress in the pathogenesis of age-related macular degeneration. *Survey of Ophthalmology*.

[2] Strauss O (2005). The retinal pigment epithelium in visual function. *Physiological Reviews*.

[3] Nowak JZ (2006). Age-related macular degeneration (AMD): pathogenesis and therapy. *Pharmacological Reports*.

[4] Tomany SC, Cruickshanks KJ, Klein R, Klein BEK, Knudtson MD (2004). Sunlight and the 10-year incidence of age-related maculopathy: the beaver dam eye study. *Archives of Ophthalmology*.

[5] Kennedy CJ, Rakoczy PE, Constable IJ (1995). Lipofuscin of the retinal pigment epithelium: a review. *Eye*.

[6] Chen H, Wiegand RD, Koutz CA, Anderson RE (1992). Docosahexaenoic acid increases in frog retinal pigment epithelium following rod photoreceptor shedding. *Experimental Eye Research*.

[7] Sparrow JR, Boulton M (2005). RPE lipofuscin and its role in retinal pathobiology. *Experimental Eye Research*.

[8] Bazan NG (2006). Survival signaling in retinal pigment epithelial cells in response to oxidative stress: significance in retinal degenerations. *Advances in Experimental Medicine and Biology*.

[9] Gao X, Talalay P (2004). Induction of phase 2 genes by sulforaphane protects, retinal pigment epithelial cells against photooxidative damage. *Proceedings of the National Academy of Sciences of the United States of America*.

[10] Gao X, Dinkova-Kostova AT, Talalay P (2001). Powerful and prolonged protection of human retinal pigment epithelial cells, keratinocytes, and mouse leukemia cells against oxidative damage: the indirect antioxidant effects of sulforaphane. *Proceedings of the National Academy of Sciences of the United States of America*.

[11] Tanito M, Masutani H, Kim Y-C, Nishikawa M, Ohira A, Yodoi J (2005). Sulforaphane induces thioredoxin through the antioxidant-responsive element and attenuates retinal light damage in mice. *Investigative Ophthalmology and Visual Science*.

[12] Kong L, Tanito M, Huang Z (2007). Delay of photoreceptor degeneration in tubby mouse by sulforaphane. *Journal of Neurochemistry*.

[13] Korn EL, Li M-C, McShane LM, Simon R (2007). An investigation of two multivariate permutation methods for controlling the false discovery proportion. *Statistics in Medicine*.

[14] Wright GW, Simon RM (2003). A random variance model for detection of differential gene expression in small microarray experiments. *Bioinformatics*.

[15] Zhou X, Li F, Kong L, Tomita H, Li C, Cao W (2005). Involvement of inflammation, degradation, and apoptosis in a mouse model of glaucoma. *Journal of Biological Chemistry*.

[16] Huang Z, Nie L, Xu M, Sun X-H (2004). Notch-induced E2A degradation requires CHIP and Hsc70 as novel facilitators of ubiquitination. *Molecular and Cellular Biology*.

[17] Nakamura H (2005). Thioredoxin and its related molecules: update 2005. *Antioxidants & Redox Signaling*.

[18] Felius J, Thompson DA, Khan NW (2002). Clinical course and visual function in a family with mutations in the RPE65 gene. *Archives of Ophthalmology*.

[19] Slomiany MG, Rosenzweig SA (2004). Autocrine effects of IGF-I-induced VEGF and IGFBP-3 secretion in retinal pigment epithelial cell line ARPE-19. *American Journal of Physiology: Cell Physiology*.

[20] Bok D, Hall MO (1971). The role of the pigment epithelium in the etiology of inherited retinal dystrophy in the rat. *Journal of Cell Biology*.

[21] Imamura Y, Noda S, Hashizume K (2006). Drusen, choroidal neovascularization, and retinal pigment epithelium dysfunction in SOD1-deficient mice: a model of age-related macular degeneration. *Proceedings of the National Academy of Sciences of the United States of America*.

[22] Talalay P, Dinkova-Kostova AT (2004). Role of nicotinamide quinone oxidoreductase 1 (NQO1) in protection against toxicity of electrophiles and reactive oxygen intermediates. *Methods in Enzymology*.

[23] Asher G, Lotem J, Sachs L, Shaul Y (2004). p53-dependent apoptosis and NAD(P)H: quinone oxidoreductase 1. *Methods in Enzymology*.

[24] Findlay VJ, Townsend DM, Morris TE, Fraser JP, He L, Tew KD (2006). A novel role for human sulfiredoxin in the reversal of glutathionylation. *Cancer Research*.

[25] Soriano FX, Baxter P, Murray LM, Sporn MB, Gillingwater TH, Hardingham GE (2009). Transcriptional regulation of the AP-1 and Nrf2 target gene sulfiredoxin. *Molecules and Cells*.

[26] Soriano FX, Léveillé F, Papadia S (2008). Induction of sulfiredoxin expression and reduction of peroxiredoxin hyperoxidation by the neuroprotective Nrf2 activator 3H-1,2-dithiole-3-thione. *Journal of Neurochemistry*.

[27] Bell KF, Al-Mubarak B, Fowler JH (2011). Mild oxidative stress activates Nrf2 in astrocytes, which contributes to neuroprotective ischemic preconditioning. *Proceedings of the National Academy of Sciences of the United States of America*.

[28] Chen Y, Shertzer HG, Schneider SN, Nebert DW, Dalton TP (2005). Glutamate cysteine ligase catalysis: dependence on ATP and modifier subunit for regulation of tissue glutathione levels. *The Journal of Biological Chemistry*.

[29] Meister A, Tate SS (1976). Glutathione and related gamma-glutamyl compounds: biosynthesis and utilization. *Annual Review of Biochemistry*.

[30] Takagi Y, Mitsui A, Nishiyama A (1999). Overexpression of thioredoxin in transgenic mice attenuates focal ischemic brain damage. *Proceedings of the National Academy of Sciences of the United States of America*.

[31] Sato A, Hara T, Nakamura H (2006). Thioredoxin-1 suppresses systemic inflammatory responses against cigarette smoking. *Antioxidants & Redox Signaling*.

[32] Tanito M, Masutani H, Nakamura H, Oka S-I, Ohira A, Yodoi J (2002). Attenuation of retinal photooxidative damage in thioredoxin transgenic mice. *Neuroscience Letters*.

[33] Muoio DM (2007). TXNIP links redox circuitry to glucose control. *Cell Metabolism*.

[34] Yamawaki H, Pan S, Lee RT, Berk BC (2005). Fluid shear stress inhibits vascular inflammation by decreasing thioredoxin-interacting protein in endothelial cells. *The Journal of Clinical Investigation*.

[35] Wang Z, Rong YP, Malone MH, Davis MC, Zhong F, Distelhorst CW (2006). Thioredoxin-interacting protein (txnip) is a glucocorticoid-regulated primary response gene involved in mediating glucocorticoid-induced apoptosis. *Oncogene*.

[36] Berndt C, Lillig CH, Holmgren A (2007). Thiol-based mechanisms of the thioredoxin and glutaredoxin systems: implications for diseases in the cardiovascular system. *American Journal of Physiology: Heart and Circulatory Physiology*.

[37] Kulinsky VI (2007). Biochemical aspects of inflammation. *Biochemistry*.

[38] Ansari AW, Bhatnagar N, Dittrich-Breiholz O, Kracht M, Schmidt RE, Heiken H (2006). Host chemokine (C-C motif) ligand-2 (CCL2) is differentially regulated in HIV type 1 (HIV-1)-infected individuals. *International Immunology*.

[39] Charo IF, Taubman MB (2004). Chemokines in the pathogenesis of vascular disease. *Circulation Research*.

[40] Hayes JD, McMahon M (2001). Molecular basis for the contribution of the antioxidant responsive element to cancer chemoprevention. *Cancer Letters*.

[41] Warabi E, Takabe W, Minami T (2007). Shear stress stabilizes NF-E2-related factor 2 and induces antioxidant genes in endothelial cells: role of reactive oxygen/nitrogen species. *Free Radical Biology & Medicine*.

