# Supplementary figures and images for: Sulforaphane Enhances the Ability of Human Retinal Pigment Epithelial Cell against Oxidative Stress, and Its Effect on Gene Expression Profile Evaluated by Microarray Analysis
Source: Oxid Med Cell Longev. 2013 Sep 25;2013:413024. doi: 10.1155/2013/413024 (PMC3800669; doi:10.1155/2013/413024)

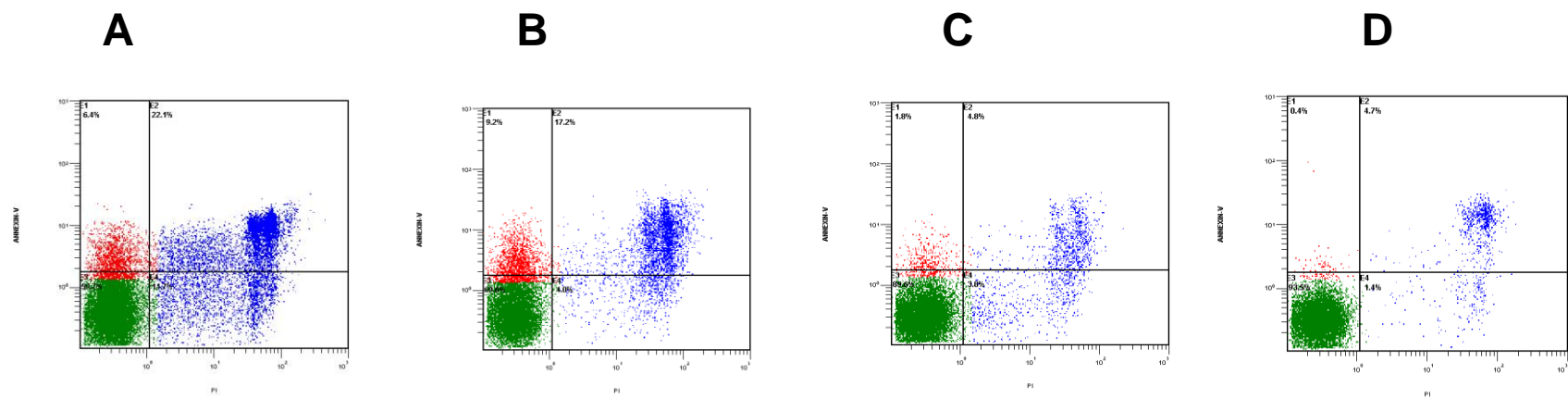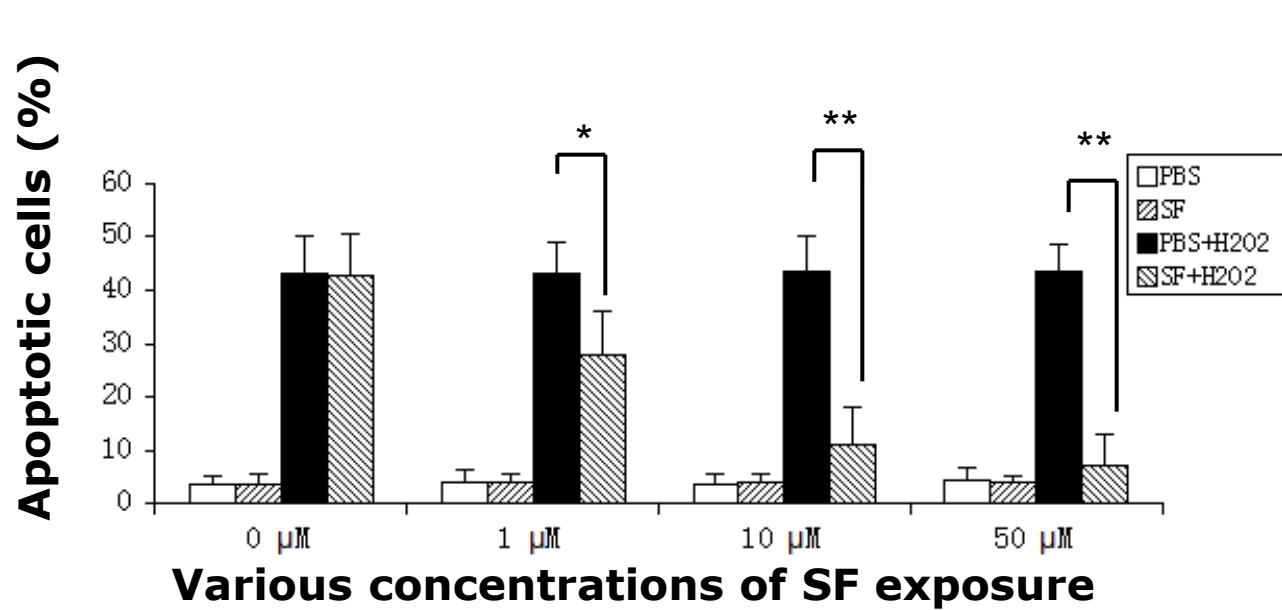

Supplementary Figure 1

Supplement: Supplementary file 1 — The antioxidative effects of different concentrations of SF in RPE 19 cells is provided in Supplementary Figure1. RPE 19 cells were pretreated with various concentrations of SF (0 µM, 1 µM, 10 µM, 50 µM, respectively) for 12 hours, then PBS or 400 µM of H2O2 were added into the cell cultures for 12 hours. Flow cytometry analysis showed that 10 µM SF was the optimal amount of SF to endow the ability of antioxidative stress for RPE 19 cells. [file 413024.f1.pdf]
